# Assessment of the Reporting Quality of Randomized Controlled Trials on Treatment of Coronary Heart Disease with Traditional Chinese Medicine from the Chinese Journal of Integrated Traditional and Western Medicine: A Systematic Review

**DOI:** 10.1371/journal.pone.0086360

**Published:** 2014-01-28

**Authors:** Fang-fang Fan, Qin Xu, Qi Sun, Sheng-jun Zhao, Ping Wang, Xue-rui Guo

**Affiliations:** 1 Department of Pharmacy, The Affiliated Traditional Medical Hospital, Xinjiang Medical University, Urumqi, Xinjiang Uygur Autonomous Region, China; 2 Medical Research Design and Data Analysis Center, The Affiliated Traditional Medical Hospital, Xinjiang Medical University, Urumqi, Xinjiang Uygur Autonomous Region, China; 3 Department of Pediatrics, The Affiliated Traditional Medical Hospital, Xinjiang Medical University, Urumqi, Xinjiang Uygur Autonomous Region, China; Macau University of Science and Technology, Macau

## Abstract

**Background:**

Due to language limitations, little is known about the reporting quality of randomized clinical trials (RCTs) on the treatment of coronary heart disease (CHD) with traditional Chinese medicine (TCM) in Chinese Journal of Integrated Traditional and Western Medicine (CJITWM).

**Objective:**

In this study, we utilized the CONSORT 2010 statement to understand the reporting quality of RCTs on CHD with TCM from the CJITWM.

**Methods:**

The China National Knowledge Infrastructure (CNKI) electronic database was searched for CJITWM RCTs on the treatment of CHD with TCM, published between Janurary 1, 2006 and December 31, 2011. We excluded articles reported as “animal studies,” “topic review,” “diagnostic test,” “editorials,” or “others.” The CONSORT checklist was applied to evaluate the reporting quality of all eligible articles by two independent authors after extensive discussion. Each item was graded as either “yes” or “no” depending on whether the authors had reported it or not.

**Results:**

We identified 21 articles meeting our inclusion criteria. The percentage of 11 of the 37 items was 4.8∼95.2%, 14 of the 37 items were reported in all included articles, while 12 items were not mentioned at all. The average reporting percentage for the “title and abstract” section was 52.4%, for the “introduction” section 100.0%, for the “methods” section 45.4%, for the “results” section 57.1%, for the “discussion” section 79.4%, and for the “other information” section 17.5%.

**Conclusion:**

In general, the reviewed RCTs were not consistent with the CONSORT 2010 statement. Authors should adhere to the CONSORT statement in reporting RCTs; editorial departments may consider the CONSORT statement as a guideline and should instruct authors to write manuscripts, and reviewers to judge them according to CONSORT statutes.

## Introduction

Overwhelming evidence shows that the current reporting quality of randomized controlled trials (RCTs) is suboptimal. Without transparent reporting, readers cannot judge the reliability and validity of trial findings nor extract information for systematic reviews. Recent methodological analyses indicate that inadequate reporting and design are associated with biased estimates of treatment effects. Such systematic error is damaging to RCTs, which are considered the gold standard for evaluating interventions because of their ability to minimize or avoid bias [1]. At present, RCTs accounted for over half of all clinical trials [2], highlighting their importance. This also reflects the standards of the Consolidated Standards of Reporting Trials (CONSORT) statement published in JAMA in 1996 [3] and the CONSORT 2010 statement published in BMJ in 2010 [1]. These guidelines consist of a checklist and flow diagram that authors can use for reporting a RCT. Many leading medical journals and major international editorial groups have endorsed the CONSORT statement, which facilitates critical appraisal and interpretation of RCTs.

Coronary heart disease (CHD) is a leading cause of death in many countries [4]. As the main treatment of CHD, modern medicine has improved dramatically in recent years. Although research of Traditional Chinese Medicine (TCM) on CHD are encouraging in many respects, the role of TCM in the prevention and treatment of CHD has been challenged due to the rapid development of modern medicine. With traditional medicinal opinions and natural medicines originating in herbs, TCM is in widespread clinical use and demonstrates a bright future in CHD treatment.

TCM, or traditional Chinese medicine, is a science of researching human physiology, pathology, diagnosis, prevention, and cure of diseases. Treatment of coronary heart disease (CHD) and preparations of Chinese medicines have more clinical applications, including Compound Salvia Pellet (CSP) for Angina Pectoris. The components of CSP are included salvia miltiorrhiza, notoginseng, and borneol, and they possess expansion of coronary artery atherosclerosis, resisting thrombosis and fall hematic fat action. CSP is the first preparation appoved by the FDA in China for the treatment of cardiovascular disease [5].

In recent years, as evidence-based medicine (EBM) has expanded, a number of multicenter RCTs with large sample sizes that focus on the prevention and treatment of CHD have been carried out in both Chinese and integrative medicine; for example, the randomized, double-blind, placebo-controlled trial on the effect of Xuezhikang (XZK) for regulating serum lipids and for the secondary prevention of CHD [6]. The XZK-treated group decreased the recurrence of nonfatal myocardial infarction in patients with CHD by 62% compared with placebo, and coronary death, coronary events, and total mortality were reduced by 45%, 30%, and 33.0%, respectively. This data filled the gap for information on the regulation of serum lipids in Oriental populations. Furthermore, proprietary Chinese medicine preparations have been used for a long time to treat CHD, with many controlled trials performed to investigate their efficacy [7]–[8].

The Chinese Journal of Integrated Traditional and Western Medicine (CJITWM) is a peer-review journal in that reflects the latest TCM research in China. However, due to language limitations, little was known about the reporting quality of this journal abroad. Although the quality of reporting in RCTs in medical sciences has been discussed, the quality of reporting in RCTs on the treatment of CHD with TCM published in the Chinese Journal of Integrated Traditional and Western Medicine (CJITWM) has not yet been assessed after publication of the CONSORT 2010 Statement. We aimed to assess the completeness of reporting of RCTs evaluating TCM in the treatment of CHD published in CJITWM from January 1, 2006 to December 31, 2011 based on the CONSORT 2010 checklist [1], and to provide recommendations for improving them in the future.

## Methods

This systematic review was performed in accordance with a protocol that prescribed search strategy, eligibility criteria, data extraction, and statistical analysis.

### Search strategy

The China National Knowledge Infrastructure (CNKI) electronic database was used to search all articles published between 2006 and 2011 that reported an RCT in which the assignment of participants to interventions was described by the words random, randomly, randomized, or randomization in the full text. We only obtained the full text of RCTs on the treatment of CHD with TCM published between 2006 and 2011. The following search strategy was used: “random, randomly, randomized, or randomization” (in full text) AND “coronary heart disease” or “angina” (in MeSH) AND “Jan. 1, 2006 to Dec. 31, 2011” AND “Chinese Journal of Integrated Traditional and Western Medicine” (in journal search tab).

### Eligibility criteria

Two reviewers independently searched the journal and selected potentially relevant articles after screening the titles and abstracts. In case of uncertain eligibility, the full text was screened. RCTs on CHD treated with TCM interventions were selected. We included RCTs in which the allocation of participants to intervention was described by the words random (randomly allocate, randomized, or randomization). We excluded trials reported as “animal studies,” “topic review,” “diagnostic test,” “editorials,” or “others.”

### Data extraction

According to the 25 items (refined to 37 items, 25 primary and 12 secondary) in the CONSORT 2010 checklist of information to include when reporting a randomized trial, we created an evaluation form for each, and for each of the 25 items in the CONSORT 2010 checklist, a “yes” (Y, scored as 1) or “no” (N, scored as 0) option was assigned depending on whether the author had reported it. Two reviewers (Fan FF, Wang P) underwent training and studying for three months on data extraction from the CONSORT 2010 statement. Two independent reviewers analyzed the data and disagreements were resolved by engaging a third expert (Xu Q or Sun Q). The data extraction items included the 25 items in the CONSORT 2010 checklist (Tables 1 and 2).

**Table 1:** The reporting number and percentage for each article of the CONSORT checklist in CJITWM.

Author	Year	Number(n)	Percentage(%)
		Yes	Yes
Guo ZB [4]	2006	14	37.8
Liu P [5]	2006	11	29.7
Lu XY [6]	2006	21	56.8
Jing L [7]	2007	17	45.9
Wei ZT [8]	2007	16	43.2
Hao Xu [9]	2007	21	56.8
Zhang Y [10]	2007	16	43.2
Wang LJ [11]	2008	19	51.4
Zhang Q [12]	2008	16	43.2
Zhang Q [13]	2008	18	48.6
Cheng WL [14]	2009	19	51.4
Hu YH [15]	2009	13	35.1
LI AM [16]	2009	13	35.1
Yan LY [17]	2009	13	35.1
Cui XY [18]	2009	18	48.6
Wu QT [19]	2009	13	35.1
Yin CH [20]	2010	15	40.5
Zhu H [21]	2010	19	51.4
Zhu HJ [22]	2010	15	40.5
Peng W [23]	2011	18	48.6
Xin L [24]	2011	19	51.4

CJITWM: Chinese Journal of Integrated Traditional and Western Medicine,

Yes: Number of trials in which the item was reported.

**Table 2:** The reporting number and percentage for each item of the CONSORT checklist in CJITWM.

Section/Topic	Item No	Checklist item	Number(n)	Percentage(%)
			Yes	Yes
Title and abstract	1a	Identification as a randomised trial in the title	1 [24]	4.8
	1b	Structured summary of trial design, methods, results, and conclusions (for specific guidance see CONSORT for abstracts)	21 [4]–[24]	100.0
Introduction				
Background and objectives	2a	Scientific background and explanation of rationale	21 [4]–[24]	100.0
	2b	Specific objectives or hypotheses	21 [4]–[24]	100.0
Methods				
Trial design	3a	Description of trial design (such as parallel, factorial) including allocation ratio	9 [6], [7], [9], [11], [13], [14], [21], [23], [24]	42.9
	3b	Important changes to methods after trial commencement (such as eligibility criteria), with reasons)	0	0
Participants	4a	Eligibility criteria for participants	21 [4]–[24]	100.0
	4b	Settings and locations where the data were collected	21 [4]–[24]	100.0
Interventions	5	The interventions for each group with sufficient details to allow replication, including how and when they were actually administered	21 [4]–[24]	100.0
Outcomes	6a	Completely defined pre-specified primary and secondary outcome measures, including how and when they were assessed	21 [4]–[24]	100.0
	6b	Any changes to trial outcomes after the trial commenced, with reasons	0	0
Sample size	7a	How sample size was determined	0	0
	7b	When applicable, explanation of any interim analyses and stopping guidelines	0	0
Randomisation				
Sequence generation	8a	Method used to generate the random allocation sequence	20 [4]–[11], [13]–[24]	95.2
	8b	Type of randomisation; details of any restriction (such as blocking and block size)	21 [4]–[24]	100.0
Allocation concealment mechanism	9	Mechanism used to implement the random allocation sequence (such as sequentially numbered containers), describing any steps taken to conceal the sequence until interventions were assigned	0	0
Implementation	10	Who generated the random allocation sequence, who enrolled participants, and who assigned participants to interventions	0	0
Blinding	11a	If done, who was blinded after assignment to interventions (for example, participants, care providers, those assessing outcomes) and how	1 [6]	4.8
	11b	If relevant, description of the similarity of interventions	6 [6], [7], [13], [14], [23], [24]	28.5
Statistical methods	12a	Statistical methods used to compare groups for primary and secondary outcomes	21 [4]–[24]	100.0
	12b	Methods for additional analyses, such as subgroup analyses and adjusted analyses	0	0
Results				
Participant flow	13a	For each, the numbers of participants who were randomly assigned, received intended treatment, and were analysed for the primary outcome group	21 [4]–[24]	100.0
	13b	For each group, losses and exclusions after randomisation, together with reasons	8 [9], [11], [12]–[14], [18], [21], [23]	38.1
Recruitment	14a	Dates defining the periods of recruitment and follow-up	19 [4], [6]–[18], [20]–[24]	90.5
	14b	Why the trial ended or was stopped	0	0
Baseline data	15	A table showing baseline demographic and clinical characteristics for each group	6 [6], [9], [12], [14], [18], [24]	28.6
Numbers analysed	16	For each group, number of participants (denominator) included in each analysis and whether the analysis was by original assigned groups	21 [4]–[24]	100.0
Outcomes and estimation	17a	For each primary and secondary outcome, results for each group, and the estimated effect size and its precision (such as 95% confidence interval)	21 [4]–[24]	100.0
	17b	For binary outcomes, presentation of both absolute and relative effect sizes is recommended)	0	0
Ancillary analyses	18	Results of any other analyses performed, including subgroup analyses and adjusted analyses, distinguishing pre-specified from exploratory	0	0
Harms	19	All important harms or unintended effects in each group (for specific guidance see CONSORT for harms	12 [4], [6], [9], [11]–[14], [18], [20], [21], [23], [24]	57.1
Discussion				
Limitations	20	Trial limitations, addressing sources of potential bias, imprecision, and, if relevant, multiplicity of analyses	8 [6], [7], [9]–[11], [14], [18], [21]	38.1
Generalisability	21	Generalisability (external validity, applicability) of the trial findings	21 [4]–[24]	100.0
Interpretation	22	Interpretation consistent with results, balancing benefits and harms, and considering other relevant evidence	21 [4]–[24]	100.0
Other information				
Registration	23	Registration number and name of trial registry	0	0
Protocol	24	Where the full trial protocol can be accessed, if available	0	0
Funding	25	Sources of funding and other support (such as supply of drugs), role of funders	11 [4], [6]–[10], [14], [18], [19], [21], [22]	52.4

CJITWM: Chinese Journal of Integrated Traditional and Western Medicine,

Yes: Number of trials in which the item was reported.

Prior to data extraction, all authors independently evaluated two RCTs reports that were not included into this study. The main content includes “CONSORT 2010 explanation and elaboration: updated guidelines for reporting parallel group randomized trials” [9], the PRISMA statement [10], and medical methodology.

### Primary and Secondary Outcomes

The primary outcome of the study was the number and percentage of applicable items on the CONSORT checklist that were reported between 2006 and 2011 in the CJITWM. The number and percentage of articles reporting each applicable section and the percentage value for each article on the checklist was the secondary outcomes.

### Statistical analyses

Data were analyzed using Microsoft Excel 2010 and SPSS software (version 19.0, IBM SPSS). In order to assess adherence to CONSORT checklist items, we calculated the number and proportion of articles describing each of the 37 items. The sum of the scores was converted to a percentage value for each article (proportion of each article = the number of reported item/37 items), each item (proportion of each item = the number of reported article/total articles), and each section (proportion of each section = the sum of items percentage of each section/total items of each section).

## Results

**Figure 1:**
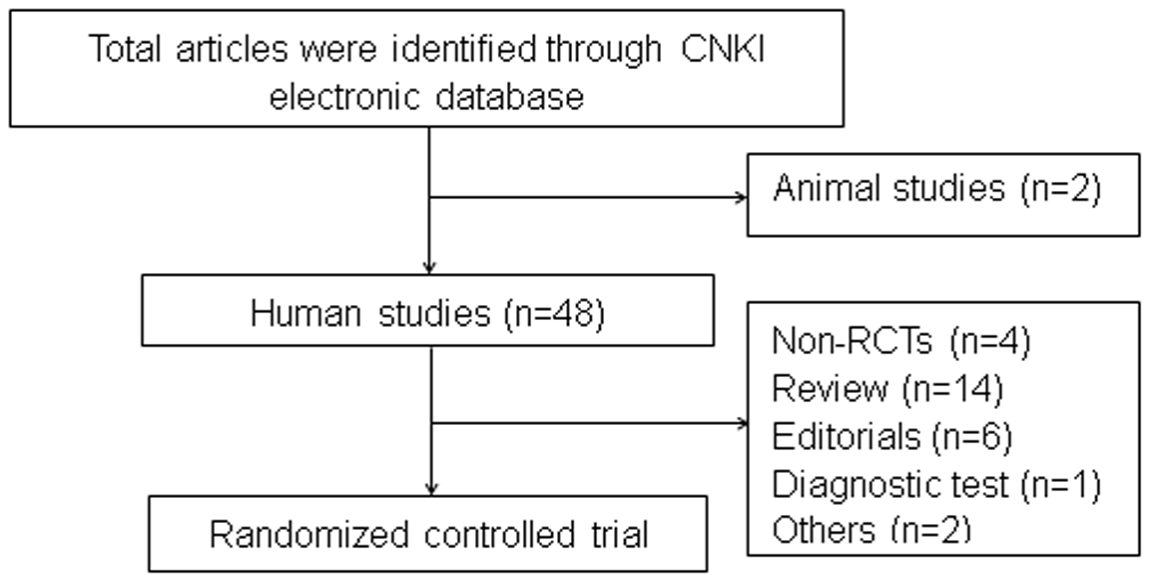
Flow diagram for the selection of articles for inclusion in systematic review.

The initial CNKI database search identified 50 records on CHD or angina published between January 1, 2006 and December 31, 2011. Of these, 2 animal studies, 14 reviews, 1 diagnostic test, 6 editorials, and 2 others were excluded. In total, 21 RCTs [11]–[31] were selected for analysis by screening the abstract and full text article. Figure 1 presents a flow chart of studies considered for inclusion.

Among the 21 included articles, according to the 37 items in CONSORT 2010 checklist, the reporting percentage in each of the articles was 29.7–56.8% (Table 1).

Among the 21 included articles, according to the 37 items in CONSORT 2010 checklist, only 4.8% (1/21) mentioned “randomization” in the title and blinding to interventions, 95.2% (20/21) reported the method used to generate the random allocation sequence, 42.9% (9/21) reported description of trial design, 28.6% (6/21) reported the similarity of interventions and baseline data with a table, 90.5% (19/21) reported dates defining the periods of recruitment and follow-up, 38.1% (8/21) reported trial limitations, losses, and exclusions after randomization, 57.1% (12/21) reported side effects, and 52.4% (11/21) reported the source of funding (Table 2). 14 of the 37 items (1b, 2a, 2b, 4a, 4b, 5, 6a, 8b, 12a, 13a, 16, 17a, 21, and 22) were reported in all included articles, while 12 items (3b, 6b, 7a, 7b, 9, 10, 12b, 14b, 17b, 18, 23, and 24) were not mentioned at all.

Table 3 summarizes the total mean number and percentages of the breakdown of scores for each section of the CONSORT checklist of the included trials. The average reporting percentage for the “title and abstract” section of the trials published in 2006 and 2011 was 52.4%: for the “introduction” section 100.0%, for the “methods” section 45.4%, for the “results” section 57.1%, for the “discussion” section 79.4%, and for the “other information” section 17.5%.

**Table 3:** The average reporting percentage for each section and the total of the CONSORT checklist in CJITWM.

Section	Number(n)	Percentage(%)
	Yes	Yes
Title and abstract	11.0	52.4
Introduction	21.0	100.0
Methods	9.5	45.4
Results	10.8	57.1
Discussion	16.7	79.4
Other information	3.7	17.5

CJITWM: Chinese Journal of Integrated Traditional and Western Medicine,

Yes: Number of trials in which the item was reported.

## Discussion

TCM has been widely applied for CHD in China. There have been a large number of controlled clinical studies published in Chinese literature, yet no systematic searching and analysis has been done in the CJITWM. In order to present these trials in an open and transparent manner, all authors need to follow widely accepted CONSORT guidelines. It is important to address this issue for many reasons. First, poorly reported clinical trials make it difficult for other researchers to assess the validity of the results, to replicate the study, and to identify gaps that need to be addressed in the design and reporting of future treatment innovations. In addition, inadequate reporting may mislead healthcare providers in their treatment decisions for patients. Lastly, policy makers depend on information provided in clinical trials to decide whether they should promote CHD with TCM to a larger population.

Although studies similar to the present study have previously been conducted, to our knowledge, this study is the first to investigate the quality of reporting of RCTs, with particular reference to CONSORT 2010 guidelines in the CJITWM following the revised CONSORT Statement (2010 version). Fan et al. [32] carried out a review on treatment of CHD with Compound Salvia Pellet (CSP) reported in 115 RCTs (up to Dec 2005) in published articles both in China and abroad. This study, which assessed the quality of the reporting of clinical trials in CHD, concluded that “the overall quality of reporting of RCTs with CSP evaluated with a revised CONSORT (2001 version) checklist was poor” [32].

Based on the CONSORT 2010 checklist, the reporting quality of RCTs on CHD in this journal was inconsistent in terminologies of the CONSORT 2010 statement. Unfortunately, despite an increasing number of RCTs assessing CHD with TCM in the past two decades, the reporting quality we reviewed is suboptimal and substantial improvement is required to meet CONSORT guidelines. Almost 50% of the trials we reviewed did not satisfy more than half of the criteria in the modified CONSORT checklist. Each of these potentially problematic areas, including title and abstract, introduction, methods, results, discussion, and other information is discussed below to help scientific readers recognize them when reviewing studies of CHD with TCM in the CJITWM and other journals.

### Title and abstract

The average reporting percentage for the “title and abstract” section of the trials published in 2006 and 2011 was 52.4%. The ability to identify a report of a randomized trial in an electronic database depends to a large extent on how it was indexed. Indexers may not classify a report as a randomized trial if the authors do not explicitly report this information [33]. A structured summary consists of trial design, methods, results, and conclusions sections. Clear, transparent, and sufficiently detailed abstracts are important because readers often assess a trial in the light of such information. Some readers use an abstract as a screening tool to decide whether to read the full article; thus, the author should adopt a structured summary to accurately report the contents of the full article. Unfortunately, we found only one article identified as a randomized trial in the title with a structured summary as well. To help ensure that a study is appropriately indexed and easily identified, authors should use the word “randomized” in the title to indicate that the participants were randomly assigned to their comparison groups.

### Introduction

The average reporting percentage for the “introduction” section was 100.0%. In practice, objectives and hypotheses are not always easily differentiated. Most reports of RCTs provide adequate information about trial objectives and hypotheses [34]. All articles reported scientific background, explanation of rationale, and specific objectives, but all introduced the study objectives without combining hypotheses, because hypotheses are more specific than objectives and are amenable to explicit statistical evaluation.

### Methods

9 articles described trial design, such as a parallel controlled trial, while others only described the trial with the word “randomized.” The word “design” is often used to refer to all aspects of how a trial is planned; it is important that researchers clearly describe these aspects of the trial, including the unit of randomization. Here we sought information on the type of trial, such as parallel group or factorial, and the conceptual framework, such as superiority or non-inferiority. All articles did not report item 3b, which may be due to the possibility that no important changes were made to the methods after trial commencement. Changes from protocols are not currently well reported. A review of comparisons with protocols showed that approximately half of journal articles describing RCTs had an unexplained discrepancy in primary outcomes [35]. All articles described eligibility criteria for participants, settings and locations where the data were collected; however, all did not introduce scale of hospitals or research institutions, such as participants recruited from primary, secondary, or tertiary health care facilities or from the community. The information on the settings and locations is crucial to judge the applicability and generalization of a trial. Thus, authors should report the number and type of settings and describe the care providers involved.

All articles described the interventions for each group with sufficient detail. Item 5 emphasized “sufficient details;” the author should describe each intervention thoroughly, including control interventions. The description should allow a clinician to use the intervention in order to know exactly when and how to apply the intervention evaluated in the trial [36].

All articles listed outcome measures, including how and when they were assessed, but most did not completely specify which were or and secondary outcome measures. The primary outcome measure is the pre-specified outcome considered to be of greatest importance and is usually the one used for sample size calculation. This item was not reported in all articles, possibly due to the lack of changes made to outcomes measures after trial commencement.

All articles did not describe how sample size was determined. Item 7a emphasized “how,” thus authors should indicate the process of sample size calculation, such as the variation of parameters, the primary outcome, and the formula of calculation or software. Sample size requires careful planning, to ensure a balance between medical and statistical considerations. Reports of studies with small sample sizes may come to an erroneous conclusion that the intervention groups do not differ, when in fact too few patients were studied to make such a claim [37]. Reviews of published trials have consistently found that a high proportion of trials have low power to detect clinically meaningful treatment effects [38]–[39]. In reality, small but clinically meaningful actual differences are much more likely than large differences but large trials are required to detect them [40]. All articles did not report this item, which may be due to no interim analyses and stopping guidelines during the trial.

20 articles described the method used to generate the allocation sequence, such as “random-number table” and “computerized random number generator,” with only 1 article reporting “randomly divided into two groups.” Item 8a emphasized “methods,” and that “randomly divided into two groups” should not substitute for “methods.” Therefore, authors should provide enough information to assess the method of sequence generation and the possibility of bias in the process of dividing into groups. All articles described the sample size of each group, but only 3 reported type of randomization and details of any restriction, such as participants allocated (1∶1). Item 8b emphasized “type,” where authors should describe restricted randomization when reporting the random method.

All articles did not describe their allocation concealment mechanism. Item 9 emphasizes “mechanism and steps to be implemented,” such as “third-party” assignment, the numbered containers, and sealed opaque envelopes. Trials in which the allocation sequence had been inadequately or unclearly concealed yielded larger estimates of treatment effects than did trials in which authors reported adequate allocation concealment. In representative samples of all randomized trials indexed on PubMed, only 18% reported any allocation concealment mechanism, but some of those that reported mechanisms were inadequate [41]. Therefore, the mechanism and steps of random allocation was a critical aspect of high quality RCTs. All articles did not describe “who generated the allocation sequence, who enrolled participants, and who assigned participants to interventions.” Item 10 emphasized “who,” i.e. who was involved the design and implement of the randomization protocol. The allocation sequence is often performed by a third-party organization without their participation in the actual clinical trials. Health workers who enrolled participants and who assigned participants to interventions did not know the random allocation sequence. Therefore, investigators must then ensure that the assignment schedule is unpredictable and locked away. Only 1 article reported who was blinded to interventions, and how this blinding was ensured. Item 11a emphasized “who” and “how,” and that authors should instead explicitly report the blinding status of people involved, for whom this may influence the validity of a trial, rather than merely describing the terms “double blind,” “single blind” or “triple blind.” A study of 200 RCTs reported as double blind found 18 different combinations of groups actually blinded when the authors of these trials were surveyed; and about one in every five of these trials—reported as double blind—did not blind participants, healthcare providers, or data collectors [42]. 6 articles described the similarity of interventions. Item 11b emphasized “similarity,” i.e., that authors should state the similarity of the characteristics of the interventions (such as appearance, taste, smell, and method of administration) so that participants and healthcare providers could not distinguish between the study drug and placebo.

All articles described statistical methods, but most used a simple description of the particular tests and analytic software used. Item 12a emphasized statistical methods of outcome measures; we suggested that authors should adopt a more structured description, for example: X1 was the primary outcome, X1 was used to analyze method A, X2 and X3 were the secondary outcomes, X2 was used to analyze method B, and X3 was used to analyze method C. Data can be analyzed in many ways, and all statistical methods may not be appropriate for all situations. It is essential to specify which statistical procedure was used for each analysis, with further clarification necessary in the results section of the report.

All articles did not describe methods for additional analyses. Some were multicenter studies with data sources and different participant demographics, so that authors should implement additional analyses. A study evaluating the reporting quality of 142 RCT full-text articles published in five leading Chinese medical journals found that only 27% described the method to generate the randomized sequence, 4% had adequate allocation concealment, and only 17% mentioned blinding [43]; these findings are supported by others in the field of TCM [44]–[45].

### Results

All articles described participant flow in some sentences, but all did not adopt a diagram to describe participant flow. 14 articles only described the numbers of participants who were randomly assigned and did not describe the numbers of participants who received the intended treatment and were analyzed for primary outcome. But from the full text, the numbers of last two phases seemed to equal the numbers of randomly assigned participants. Item 13a told authors that the numbers of participants may change in the three stages, including design, study, and analysis. Although CONSORT strongly recommends using this graphical device to communicate participant flow throughout the study, there is no specific, prescribed format. Participant flow was not necessary for some RCTs, if design and execution were straightforward, particularly if there were no losses to follow up or exclusions. However, in more complex studies, authors should adopt the flow for readers to discern whether and why some participants did not receive the allocated treatment, were lost to follow-up, or were excluded from the analysis. 8 articles described losses and exclusions after randomization with reasons for each group. Item 13b emphasized “numbers and reasons.” Authors should have described the numbers of design phase, and the numbers and reasons for excluding participants from the analysis. Only in that way, some protocol deviations may be reported, such as participants who did not receive the intended intervention. 19 articles described “dates defining the periods of recruitment and follow-up.” Knowing when a study took place and over what period participants were recruited places the study in historical context. The length of follow-up is not always a fixed period after randomization. In many RCTs in which the outcome is time to an event, follow-up of all participants is ended on a specific date. All articles did not describe why the trial ended or was stopped, which may be because there was no such situation. If so, authors should indicate why the trial came to an end and also disclose extrinsic factors that affected the decision to stop the trial, and who made the decision to stop the trial, including reporting the role the funding agency in the deliberations and decision to stop the trial [46]. All articles described baseline data to some degree, while only 6 articles showed baseline data with a table. Although the eligibility criteria indicated who was eligible for the trial, it is also important to know the characteristics of the participants who were actually included. This information allows readers, especially clinicians, to judge how relevant the results of a trial might be to an individual patient. Thus, author should describe this baseline information in a table.

All articles described number of participants (denominator) included in each analysis, but only 2 articles mentioned intention-to-treat (ITT) analysis. The meaning of the ITT analysis was that we should include all randomized participants in the analysis, and all retained in the group to which they were allocated in order to fully preserve the benefit of randomization. The conditions defined an ITT analysis, which was widely recommended as the preferred analysis strategy [47]–[48]. ITT analysis is generally favored because it avoids bias associated with non-random loss of participants [49]–[51]. Regardless of whether authors use the term “ITT,” they should clarify which and how many participants are included in each analysis. Non-compliance with assigned therapy may mean that the intention-to-treat analysis underestimates the potential benefit of the treatment, so that additional analyses, such as a per-protocol analysis, may be considered [52]–[53]. It should be noted, however, that such analyses are often considerably flawed [54].

All articles described results for each group, but only 1 article reported the 95% confidence interval. For each outcome, study results should be reported as a summary of the outcome in each group, together with the contrast between the groups, known as the effect size. Furthermore, for all outcomes, authors should provide a confidence interval to indicate the precision (uncertainty) of the estimate. Many journals require or strongly encourage the use of confidence intervals [55]. They are especially valuable in relation to differences that do not meet conventional statistical significance, for which they often indicate that the result does not rule out an important clinical difference. The use of confidence intervals has increased markedly in recent years, although not in all medical specialties [56]. All articles did not mention binary outcome, which may be due to their unavailability. If so, both the relative effect (risk ratio [relative risk] or odds ratio) and the absolute effect should be reported (with confidence intervals), as neither the relative measure nor the absolute measure alone gives a complete picture of the effect and its implications. The common absolute effect included the numerator, denominator, and effect difference. The common relative effect included the risk ratio or risk difference. For survival time data, it could be the hazard ratio or difference in median survival time.

All articles did not describe subgroup analyses and adjusted analyses. Some of the 21 RCTs were multicenter studies with large differences in data sources and participant demographic data. It would be inappropriate to statistically analyze hospitals or research institutions together. In fact, researchers should pre-specify subgroup analyses of other hospital or research institutions, because it may influence the calculation of sample size.

12 articles described adverse effects. The implication of reporting adverse effects is that it easily enables the reader to understand information about the risks and benefits of interventions to make rational and balanced decisions. A review of trials published in six general medical journals in 2006 to 2007 found that, although 89% of 133 reports mentioned adverse events, no information on severe adverse events and withdrawal of patients due to an adverse event was given in 27% and 48% of articles, respectively [57].

### Discussion

8 articles described trial limitations, but were superficial and incomplete. Common limitations included: not reporting how sample size was determined, allocation concealment mechanism and implementation, and ITT analysis. These reasons indicated that internal validity was unreliable. While all articles did not report external validity of the trial findings in the discussion section, all described eligibility criteria for participants, settings and locations where the data were collected, interventions, and outcomes, in the materials and methods sections. External validity, also called generalization or applicability, is the extent to which the results of a study can be generalized to other circumstances [58]. Internal validity, the extent to which the design and conduct of the trial eliminate the possibility of bias, is a prerequisite for external validity; the results of a flawed trial are invalid and the question of its external validity becomes irrelevant. All articles described interpretation consistent with results and compared with other articles. Readers will want to know how the present trial's results relate to those of other RCTs. This can best be achieved by including a formal systematic review in the results or discussion section of the report [59]–[60].

### Other information

All articles did not describe registration number and name of trial registry. The World Health Organization states that “the registration of all interventional trials is a scientific, ethical, and moral responsibility.” By registering a randomized trial, authors typically report a minimal set of information and obtain a unique trial registration number. If authors had not registered their trial they should explicitly state this and give the reason. All articles did not describe “where the full trial protocol can be accessed, if available.” A protocol for the complete trial is important because it pre-specifies the methods of the randomized trial, such as primary outcome. Having a protocol can help to restrict the likelihood of undeclared *post hoc* changes to the trial methods and selective outcome reporting.

There were several ways to obtain the trial protocol. Journals reporting a trial's primary results can make the trial protocol available on their web site. Accessibility to the trial results and protocol is enhanced when the journal is open access. Some journals (such as trials) publish trial protocols, and such a publication can be referenced when reporting the trial's principal results. Trial registration will also ensure that many trial protocol details are available. Trial investigators may also be able to post their trial protocol on a website through their employer. The issue is that most investigators were not aware of publicizing the trial protocol. Certainly, it cannot exclude someone with no complete trial protocol.

11 articles described sources of funding, but all did not introduce the role of funders. Authors should report the sources of funding for the trial, as this is important information for readers assessing it. The level of involvement by a funder and their influence on the design, conduct, analysis, and reporting of a trial varies. If the funder had no such involvement, the authors should state this fact.

### Study Limitations

Our study has several limitations. First, we did not contact authors when items were not reported because authors may not have adhered to CONSORT 2010 for reporting RCTs, with some items performed in their studies not reported in the articles. Second, we assessed each item with a “yes” or “no” response according to whether the author had reported all the contents listed in the refined items. A better way to show integrity and accuracy of RCTs would have been to have used a quantitative score approach to assess each article. A study adopted the visual analogue scale (VAS) approach to evaluate the overall quality of the report, “Clinical study of efficacy of GnRH-α combined with add back therapy in treatment of endometriosis” [61]. A study adopted five levels (adequately standardized, relatively standardized, inadequately standardized, not standardized, and not reported) to evaluate the overall quality of the report “Evaluation of the paper titled “Application of Tumor Type M2 Pyruvate Kinase in Diagnosis of Lung Cancer” based on the STARD statement”[62]. This method also can assess reporting quality of RCTs with the CONSORT 2010 statement. Third, we did not use the CONSORT for TCM to evaluate the reporting quality of RCTs on CHD in the journal. CONSORT for TCM is based on the CONSORT 2010 statement [63], with some items (background, objective, participants, intervention and outcomes, baseline, ancillary analyses, interpretation) adding related content of TCM. We selected the CONSORT 2010 statement instead of CONSORT for TCM to assess these articles of TCM because CONSORT for the TCM checklist did not include limitations, registration, protocol, funding, and secondary items. These items also reported the integrity and accuracy of RCTs. Fourth, we did not calculate the Kappa statistic to quantitatively measure inter-observer agreement.

Despite these, our study has some strengths. We conducted an objective data extraction process, with the domains that were included in RCTs marked as “yes” and those not reported as “no” based on standard checklist items recommended by the CONSORT 2010 statement without reviewer inference. Thus, this study's methodology is reproducible.

## Conclusion

We demonstrated that the reviewed RCTs were generally not consistent with the CONSORT 2010 statement. Many studies proved that the use of the CONSORT statement has demonstrable benefits in improving the reporting quality of RCTs [64]. Thus, we suggest that authors should adhere to the CONSORT 2010 statement in reporting RCTs, and that editorial departments may consider the CONSORT statement as a guideline and instruct authors to draft manuscripts, and reviewers to judge manuscripts, according to the CONSORT statement, in order to further improve the reporting quality of RCTs.

## Supporting Information

Checklist S1 **PRISMA checklist.** PRISMA 2009 checklist of information to include when reporting a systematic review.
(DOC)
